# Predictors of Response to Occipital Nerve Stimulation in Patients with Refractory Chronic Cluster Headache: Protocol for a Prospective Observational Study

**DOI:** 10.3390/brainsci16030256

**Published:** 2026-02-25

**Authors:** Leonardo Portocarrero-Sánchez, Alfonso Gil-Martínez, José Francisco Paz-Solís, María Román-Aragón, Beatriz Mansilla-Fernández, Ignacio Elizagaray-García, Cristian Rizea, Saúl Marín-Esteban, Cristina Utrilla, Celia María de-Toro-Cañizares, Lucía Zaballa-Pérez, Rebeca Gallego-Ruiz, Maria José Ruiz-Castrillo, Javier Díaz-de-Terán

**Affiliations:** 1 Neurology Department, University Hospital La Paz, 28046 Madrid, Spain; leonardo9493@gmail.com (L.P.-S.);; 2 CranioSPain Research Group, Centro Superior de Estudios Universitarios La Salle, Universidad Autónoma de Madrid, 28023 Madrid, Spain; 3 Departamento de Fisioterapia, Centro Superior de Estudios Universitarios La Salle, Universidad Autónoma de Madrid, 28023 Madrid, Spain; 4 Departamento de Fisioterapia, Hospital Universitario La Paz-Carlos III, IdiPAZ, 28046 Madrid, Spain; 5 Neurosurgery Department, University Hospital La Paz, 28046 Madrid, Spain; 6 Radiology Department, University Hospital La Paz, 28046 Madrid, Spain; cristinautrilla@hotmail.com; 7 Fisioterapia Balance, 28029 Madrid, Spain; 8 Hospital La Paz Institute for Health Research—IdiPAZ (La Paz University Hospital—Universidad Autónoma de Madrid), 28046 Madrid, Spain

**Keywords:** cluster headache, chronic, refractory, rCCH, occipital nerve stimulation, ONS, neuromodulation

## Abstract

**Highlights:**

**What are the main findings?**

- This prospective study protocol introduces a multidimensional assessment panel—including structural magnetic resonance imaging (MRI), serum neuropeptides (calcitonin gene-related peptide (CGRP), pituitary adenylate cyclase-activating peptide 38 (PACAP38), vasoactive intestinal peptide (VIP)), neuropsychological evaluation, auditory evoked potentials, and quantitative sensory testing—to identify predictors of response to occipital nerve stimulation (ONS) in refractory chronic cluster headache.
- Transcutaneous electrical nerve stimulation (TENS) is evaluated as a potential non-invasive preoperative screening tool to predict ONS outcomes.

**What are the implications of the main findings?**

- Identification of reliable predictive biomarkers would enable precision patient selection for ONS, reducing unnecessary surgical complications and optimizing healthcare resource allocation.
- This multidimensional approach may also help us understand the biological basis of ONS response mechanisms and serve as a starting point for larger validation studies.

**Abstract:**

**Background**: Occipital nerve stimulation (ONS) is an effective therapy for patients with refractory chronic cluster headache (rCCH); however, it is not without complications, and to date, there are no conclusive findings regarding factors that would allow the prediction of treatment response. The primary objective of this study is to identify such factors to improve patient selection. **Methods**: This single-center prospective observational study will be conducted at the Department of Neurology, Hospital Universitario La Paz (Madrid, Spain). Given the low prevalence of rCCH, a convenience sampling approach will be adopted, with an expected enrollment of a minimum of 15 patients over 24 months of the study. The study is structured into three periods: Pre-ONS (pre-implantation), ONS (implantation), and Post-ONS (follow-up at 12 months). During the pre-implantation phase, patients will undergo a multidimensional assessment encompassing structural 3T brain magnetic resonance imaging (MRI), blood analysis (calcitonin gene-related peptide (CGRP), pituitary adenylate cyclase-activating peptide 38 (PACAP38), and vasoactive intestinal peptide (VIP)), neuropsychological evaluation, auditory evoked potentials, algometry (pressure pain threshold, temporal summation, conditioned pain modulation), and transcutaneous electrical nerve stimulation (TENS). Follow-up visits will be conducted at 3, 6, and 12 months post-implantation. **Results**: This study aims to identify biomarkers or their combinations capable of reliably predicting patients who would benefit from ONS. **Conclusions**: Through this multidimensional assessment, this study seeks to identify predictive factors of response to ONS, thereby improving patient selection, optimizing healthcare resources, and advancing the understanding of treatment response mechanisms.

## 1. Introduction

Cluster headache (CH) is a primary headache disorder characterized by bouts of high-intensity unilateral pain attacks lasting 15 to 180 min, with a frequency of up to 8 attacks per day [1]. Its prevalence is estimated to be 124 per 100,000 inhabitants, with an annual incidence of 53 new cases per 100,000 [2]. The estimated annual cost of these patients to public healthcare systems reaches €21,000 [3].

Approximately 20% of CH cases become chronic (chronic cluster headache (CCH)), with pain-free periods between bouts of less than 3 months [1]. According to the European Headache Federation (EHF) criteria [4], when a patient with CCH fails to respond to three conventional pharmacological therapeutic trials and continues to experience at least three weekly attacks with an impact on quality of life, the condition is classified as refractory CCH (rCCH). Approximately 10% of patients with CCH present with refractory forms, experiencing an even greater degree of disability [5].

Occipital nerve stimulation (ONS) is a minimally invasive neuromodulation technique recommended for the management of rCCH [6,7]. From a pathophysiological standpoint, positron emission tomography studies have demonstrated that ONS normalizes hypermetabolic cortical areas involved in pain processing [8].

The ICON clinical trial [9], which included 150 patients, demonstrated a significant reduction in attack frequency and intensity, and its 8-year extension [10] confirmed its long-term efficacy and safety. Together with other studies published in the literature, the effectiveness has been estimated to range between 40% and 90%, with a mean of 57% [7]. Adverse effects, although mild and less frequent than those of more invasive techniques such as deep brain stimulation, have been reported in up to 70% of cases [7]. Consequently, the European Academy of Neurology guidelines do not currently issue a strong recommendation for this procedure; however, they explicitly acknowledge that approximately 50% of patients suffering from this devastating condition achieve significant clinical improvement, highlighting a critical need for refined patient selection criteria [11].

Treatment costs are estimated at €28,186 per case; however, cost-effectiveness studies show a mean annual saving of €1344 per patient compared with conventional therapy, adding 0.28 quality-adjusted life years [12].

Despite these promising results, a significant percentage of patients require explantation due to low effectiveness of the treatment. According to the CLUSTER-MAD registry, of the 26 patients implanted in the Community of Madrid, 50% were explanted due to lack of response [13]. This study suggested that an early age of onset, smoking, and nocturnal and seasonal exacerbations were associated with a lack of response to this treatment. Most studies, including the ICON trial, have unsuccessfully attempted to identify response predictors [9]. Furthermore, there is limited evidence suggesting that high baseline anxiety and C2–C3 pain may be negative predictors [14], whereas the response to transcutaneous electrical nerve stimulation (TENS) may be a positive predictor [15].

In this context, the pathophysiology of CH involves alterations across multiple biological domains, which provides a rationale for a multidimensional approach to identifying predictive biomarkers of treatment response.

At the structural level, recent neuroimaging investigations have demonstrated increased bilateral anterior hypothalamic volume in patients with CH, particularly in the suprachiasmatic and paraventricular nuclei [16], suggesting that morphometric changes in key regions of the trigeminovascular and circadian systems may reflect disease severity or treatment susceptibility. At the somatosensory level, our group has published research on central sensitization in CH, finding lower pressure pain thresholds (PPT) and a greater degree of allodynia in patients with CH compared to controls [17], indicating that quantitative sensory testing may capture the degree of central sensitization relevant to neuromodulation outcomes. Complementarily, neurophysiological studies have shown that the amplitude of auditory evoked potentials is significantly lower in patients with CH both during and outside of bouts [18], which may reflect altered cortical excitability and habituation patterns that could influence the response to ONS.

Beyond these neurobiological dimensions, the neuropsychological profile of patients with CH is characterized by a high comorbidity with psychiatric disorders, including depression, anxiety, substance use disorders, and personality disorders [19]. Our group has found higher levels of catastrophizing, depression, and anxiety in patients with CH than in controls [20,21], factors that may modulate treatment expectations and adherence, and ultimately influence therapeutic outcomes. At the molecular level, neuropeptides of the trigeminovascular system, such as calcitonin gene-related peptide (CGRP), pituitary adenylate cyclase-activating peptide 38 (PACAP38), and vasoactive intestinal peptide (VIP), are implicated in the pathophysiology of CH [22]. CGRP infusion has been shown to precipitate attacks in 50% of patients with CCH [23]; however, recent studies have found that CGRP levels do not differ between clinical subtypes and are decreased compared to those in controls [24], highlighting the need for further investigation of these biomarkers in the context of treatment response prediction.

Finally, from a clinical screening perspective, a case series of 41 patients with refractory headache described a favorable response to ONS in those with a prior favorable response to transcutaneous electrical nerve stimulation (TENS) [15], although anesthetic blockade of the greater occipital nerve did not predict response in a series of 7 patients [25]. According to the HortONS study, TENS is evaluated as a feasible preoperative screening tool for ONS outcomes by comparing the effect of attack prevention of TENS and tonic ONS [26].

To date, no single biomarker has been shown to reliably predict ONS response in patients with rCCH. Through a multidimensional evaluation integrating structural, neurophysiological, somatosensory, neuropsychological, molecular, and clinical domains, this project aims to develop a predictive biomarker panel that enables appropriate patient selection, avoiding unnecessary complications and advancing toward precision medicine.

## 2. Materials and Methods

### 2.1. Participants

We will include patients who met the following criteria: aged ≥ 18 years, diagnosed with CCH based on the International Classification of Headache Disorders 3rd Edition and rCCH based on the European Headache Federation criteria of 2014, as determined by a neurologist with expertise in headache disorders, and consent to receive surgical treatment with ONS according to clinical practice guidelines.

The exclusion criteria are: psychiatric or cognitive pathology precluding comprehension of informed consent or hindering follow-up, carriers of cardiac pacemakers or other neuromodulation devices, prior surgery on the posterior cranial fossa or C2–C3 nerve roots, epilepsy or contraindication for electrical stimulation devices, and pregnancy or desire for pregnancy. Since this is an observational study reflecting routine clinical practice (as specified in the study design section), no prior or concomitant medical treatment was considered as an exclusion criterion.

### 2.2. Study Design

This single-center prospective observational study will be conducted at the Department of Neurology, Hospital Universitario La Paz (Madrid, Spain).

The study was approved by the Research Ethics Committee for Medicinal Products (CEIm) of the Hospital Universitario La Paz (PI-6215). All participants will provide written informed consent prior to their enrollment. All procedures will be conducted in accordance with the Declaration of Helsinki, Good Clinical Practice Guidelines, and applicable data protection regulations (Spanish Law 14/2007 on Biomedical Research, EU General Data Protection Regulation 2016/679, and Spanish Organic Law 3/2018 on Personal Data Protection).

### 2.3. Sample Size

Given the low prevalence of rCCH (approximately 12.4 per 100,000 inhabitants), we adopted a convenience sampling approach. Based on clinical practice data, a minimum of 15 patients will be expected to be enrolled over a 24-month period.

### 2.4. Protocol

**Figure 1:**
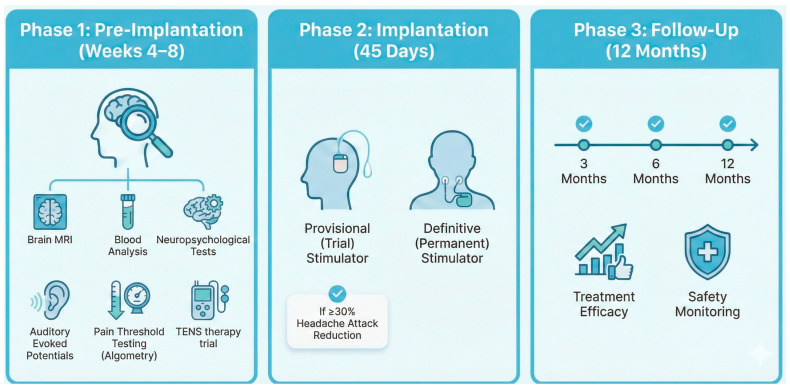
Study Design.

The study is structured into three sequential periods (Figure 1): Pre-ONS (pre-implantation assessment), ONS (surgical implantation), and Post-ONS (follow-up). The procedures performed during each period are described below and summarized in Table 1.

#### 2.4.1. Period 1: Pre-ONS (Pre-Implantation, Weeks 0–8)

This period encompasses the screening visit and the baseline multidimensional assessment of all study biomarkers. All evaluations described in this section are performed exclusively during this pre-implantation phase.

***Screening Visit (Day 0)***

At the screening visit, informed consent is obtained, and the following data are collected: demographic information, complete medical and headache history (including time since onset, time of diagnosis and time since chronification), and details of prior and current treatments. Any changes in preventive medication during the study period are systematically recorded. Changes in concomitant preventive medication during the study period will be systematically recorded. Inclusion and exclusion criteria are verified, and eligible participants are provided with an electronic headache diary (eDiary) linked to the Research Electronic Data Capture (REDCap) platform via QR code. From this point onward, patients electronically record attack frequency (per day/week), intensity, duration, use of rescue medication (triptans or oxygen, as per their usual treatment), and adverse effects. The use of an electronic diary with prospective real-time data entry is expected to minimize recall bias and improve the accuracy of patient-reported outcomes compared with retrospective paper-based methods.

Additionally, the following patient-reported outcome measures are administered at baseline: the EuroQoL five-dimension questionnaire (EQ-5D) to assess health-related quality of life, the Cluster Headache Quality of Life questionnaire (CHQ) as a disease-specific quality of life measure, the Headache Impact Test-6 (HIT-6) to quantify headache-related disability, and the Allodynia Symptom Checklist-12 (ASC-12) to evaluate the presence and severity of cutaneous allodynia. These instruments are re-administered at each follow-up visit (3, 6, and 12 months) to assess longitudinal changes.

***Multidimensional Assessment (Weeks 4–8)***

During the pre-implantation period, the following assessments are conducted on separate days:

**(a)**  **Structural 3T Brain Magnetic Resonance Imaging (MRI).**

Structural brain MRI (General Electric Signa (Madrid, Spain) with 3T field) with a head coil will be performed prior to treatment. Standard sequences required to exclude underlying pathologies or secondary causes of headache include T1-weighted, T2/fluid attenuation inversion recovery (FLAIR), susceptibility-weighted imaging (SWI), and diffusion-weighted imaging (DWI). The study protocol will include axial sequences comprising T2-turbo spin echo (TSE, 1:53 min), FLAIR (2:42 min), DWI (2:29 min), and SWI (4:16 min, particularly sensitive for detecting microhemorrhages). Additionally, a sagittal 3D T1-weighted sequence (5:26 min) will be acquired for anatomical definition and capacity to generate multiplanar reconstructions. For axial sequences, a slice thickness of 5 mm will be selected with 10% interslice spacing and angled conventionally along the imaginary line connecting the anterior and posterior white commissures (AC-PC line). The 3D T1-weighted sequence will be used for volumetric and cortical thickness analyses using FreeSurfer (version 8.1.0)/FMRIB Software Library (FSL) software (version 6.0.7). Regions of interest will include anterior and posterior hypothalamus, amygdala, and cerebellum volumes, as well as cortical thickness of the primary sensory cortex, orbitofrontal cortex, and anterior cingulate cortex. Estimated duration: 25 min.

**(b)**  **Blood Sample.**

Blood samples will be collected and processed (centrifugation and storage) by specialized nursing staff from the unit. Subsequent determination of CGRP, PACAP38, and VIP levels using commercially validated enzyme-linked immunosorbent assay (ELISA) kits (Abbexa, Cambridge, UK) will be performed by trained laboratory personnel at the research unit (IdiPAZ).

**(c)**  **Neuropsychological Assessment.**

A 120 min evaluation will be conducted by a neuropsychologist using a battery of validated instruments including the Minnesota Multiphasic Personality Inventory-3 (MMPI-3) [20], Big Five Personality Questionnaire (MASK-5) [27], Hospital Anxiety and Depression Scale (HADS) [28], and Pain Catastrophizing Scale (PCS) [29]

**(d)**  **Auditory Evoked Potentials.**

Although previous research has demonstrated altered cortical auditory evoked potentials in patients with CH [18], the present study employs brainstem auditory evoked responses (BAER) to assess the functional integrity of the auditory pathway at the brainstem level [30]. This choice is determined by both practical and pathophysiological considerations. From a practical standpoint, the neurophysiology equipment available at our center is configured to perform BAER, which constitutes the standard auditory evoked potential technique in routine clinical neurophysiology. From a pathophysiological perspective, the brainstem harbors key structures implicated in CH—including the trigeminal nucleus caudalis, locus coeruleus, and periaqueductal gray—that are anatomically contiguous with the auditory relay nuclei assessed by BAER (cochlear nucleus, superior olivary complex, lateral lemniscus, and inferior colliculus). Furthermore, ONS is hypothesized to exert its therapeutic effect through modulation of brainstem circuits via trigeminocervical afferents, making brainstem-level neurophysiological assessment particularly relevant as a potential predictor of treatment response. Additionally, BAER represents a highly reproducible and standardized technique suitable for a multicenter-scalable protocol.

Both auditory nerves will be evaluated individually through monaural click-type stimuli. A standard vertex-to-ipsilateral mastoid electrode montage (active electrode at Cz, reference at ipsilateral mastoid, ground at forehead) will be used, with electrode impedances maintained below 5 kΩ. Click stimuli of approximately 0.1 ms duration will be presented at a rate of 10/s, using rarefaction or alternating polarity. Stimulus intensity will range from 30 dB to 80–90 dB nHL, with contralateral masking applied at up to 60 dB. A bandpass filter of 100–3000 Hz will be applied, with a 10 ms analysis epoch. A minimum of 1000–2000 sweeps will be averaged per recording to ensure an adequate signal-to-noise ratio. Waves I, III, and V will be identified, and the following variables will be extracted: absolute latencies of Waves I, III, and V; interpeak intervals (I–III, III–V, I–V); and interaural latency differences. All values will be compared against the laboratory’s age- and equipment-matched normative dataset. Estimated duration: 30 min.

**(e)**  **Algometry Study.**

This assessment will be performed by specialized physiotherapists with extensive clinical and research experience in these procedures. Measurements will be obtained in the trigeminal and extratrigeminal regions, such as the tibia, following a validated protocol previously employed by our research group. Gradually increasing pressure will be applied at a rate of 50 kPa/s, using a digital algometer (Wagner Instruments, Greenwich, CT, USA). Participants will be instructed to respond with a verbal cue when the pressure sensation generated by the algometer becomes painful. The order of evaluation will be randomized for each participant. Data will be recorded in kg/cm^2^.

*Temporal summation of stimuli.*

The trigeminal region will be evaluated using a 128 mN pinprick instrument, whereas the 256 mN instrument (MRC Systems, Heidelberg, Germany) will be used for the extratrigeminal region. Pain intensity perceived following a single stimulus will be quantified using a visual analog scale (VAS). Subsequently, 10 repeated stimuli will be applied at the same location with the same intensity, with a 1 s interstimulus interval. Pain intensity perceived following the last of the 10 stimuli will be quantified using the VAS. The magnitude of temporal summation will be calculated as the difference between the VAS score induced by the last of the 10 stimuli and the VAS score induced by the first independently administered stimulus.

*Conditioned pain modulation (CPM).*

Participants will be positioned seated for the first test stimulus, which will be recorded as the mean pressure pain threshold (PPT) at different points in the trigeminal area (V1, V2) and extratrigeminal area (C5 and tibialis anterior muscle) ipsilateral to headache. Subsequently, a conditioning thermal stimulus will be administered by immersing the hand contralateral to the headache in a cold pressor device (10–12 °C) for 2 min. The second test stimulus (PPT) will be obtained immediately after the cold conditioning stimulus, and PPT values will be recorded again in the trigeminal (V1, V2) and extratrigeminal (C5 and tibialis anterior muscle) areas ipsilateral to the headache. The modulation effect will be calculated as the difference between the second and first PPT measurements for each area, while the percentage change will be obtained as follows: percentage change = (PPT second test − PPT first test)/PPT first test.

**(f)**  **Transcutaneous Electrical Nerve Stimulation (TENS).**

TENS will be delivered using a NeuroTrac MultiTens device (Verity Medical Ltd., Braishfield, UK), a dual-channel digital stimulator with an asymmetric biphasic rectangular constant-current waveform (amplitude: 0–90 mA in 0.5 mA increments; frequency: 2–200 Hz; pulse width: 50–450 μs). Two self-adhesive electrodes (50 × 50 mm) will be placed bilaterally over the greater occipital nerve, at the level of the external occipital protuberance (inion), approximately 3–4 cm from the midline, following the protocol described by Fogh-Andersen et al. [26,31]. The device will be pre-programmed by a physiotherapist with expertise in neuromodulation in gate control mode (continuous stimulation; frequency: 80 Hz; pulse width: 100 μs), and subsequently locked to prevent parameter modification by the patient. Patients will self-administer three 30 min sessions per day at home during the pre-implantation period. Treatment compliance will be objectively monitored through the device’s built-in data logger, which records up to five daily sessions over 60 consecutive days, allowing the research team to review adherence at follow-up visits. Response to TENS will be defined as a ≥30% reduction in weekly attack frequency during the TENS treatment period compared with the baseline recording period, consistent with the response threshold applied for ONS assessment in this protocol. In addition, the percentage change in attack frequency during the TENS period will be analyzed as a continuous predictor of subsequent ONS response, to explore potential dose–response relationships.

#### 2.4.2. Period 2: ONS (Implantation, Weeks 8–14)

This period comprises the surgical implantation of the ONS system in two stages.

ONS Visit 1—Trial Electrode Implantation (Week 8).

A trial electrode (provisional ONS) is implanted according to the standardized neurosurgery protocol of our center, which has remained consistent for 15 years [32].

ONS Visit 2—Definitive Generator Implantation (Week 14).

Patients demonstrating a ≥30% reduction in weekly attack frequency (monitored via eDiary) during the trial period will undergo definitive implantable pulse generator (IPG) placement. Patients who do not meet this threshold will have the trial electrode explanted.

No additional biomarker assessments are performed during this period. Clinical data (attack frequency, intensity, duration, rescue medication use, and adverse effects) continue to be recorded by the patient via the eDiary throughout this phase.

#### 2.4.3. Period 3: Post-ONS (Follow-Up, 12 Months)

Follow-up visits are scheduled at 3, 6, and 12 months after definitive generator implantation. At each visit, the following clinical parameters are evaluated: weekly attack frequency, attack intensity (VAS), attack duration, use of rescue medication, adverse effects, and treatment response defined as ≥30% reduction in weekly attack frequency compared with baseline. The Patient Global Impression of Change (PGI-C) scale is also administered at each follow-up visit.

In addition, the following patient-reported outcome measures are administered at each follow-up visit: EQ-5D, CHQ, HIT-6, ASC-12 and HADS.

Algometry assessments (pressure pain threshold, temporal summation, and conditioned pain modulation), following the same protocol described in the pre-implantation phase, are repeated at 3, 6, and 12 months to evaluate longitudinal changes in somatosensory processing. Blood samples for determination of CGRP, PACAP38, and VIP levels are collected at 3 months post-implantation to assess early biochemical changes associated with treatment response.

No reassessment of structural brain MRI, neuropsychological evaluation, auditory evoked potentials, or TENS is performed during the follow-up period. Throughout the entire study period, patients continue recording clinical variables on their eDiary via the REDCap platform. If a patient experiences a pain attack, they are permitted to use their usual rescue medication.

### 2.5. Data Analysis

Statistical analysis will be performed using R software (version 4.3.1 or higher) (R Foundation for Statistical Computing, Vienna, Austria). Given the exploratory nature of this prospective study and the sample size constraints (N < 30), a rigorous statistical framework will be applied to maximize the validity of the findings.

#### 2.5.1. Descriptive Statistics and Bivariate Analysis

Normality of distributions will be assessed using the Shapiro–Wilk test, which is robust for small sample sizes. Categorical variables will be reported as frequencies and percentages. Quantitative variables will be expressed as means with standard deviations (SD) for normally distributed data, or medians with interquartile ranges (IQR) for non-parametric data.

Bivariate comparisons between responders (defined as ≥30% reduction in weekly attack frequency at 12 months) and non-responders will be conducted using Fisher’s exact test for categorical variables. For continuous variables, Student’s *t*-test or the Mann–Whitney U test will be employed depending on the distribution assumptions. The significance level will be set at α < 0.05 for initial screening.

To account for the simultaneous analysis of multiple biomarkers and minimize the risk of Type I error inflation, we will apply the Benjamini–Hochberg procedure to control the false discovery rate (FDR) at q < 0.10. This correction will be applied across all individual bivariate tests performed (estimated at approximately 15–20 comparisons, corresponding to the candidate biomarkers derived from the six assessment domains: structural neuroimaging, serum neuropeptides, neuropsychological scales, auditory evoked potentials, algometry measures, and TENS response). This method was selected over more conservative approaches such as Bonferroni correction, as it provides a better balance between Type I error control and statistical power in exploratory studies with limited sample sizes. In addition, the penalized regression framework employed in the multidimensional modeling phase (Section 2.5.2) inherently addresses multiplicity through coefficient shrinkage and automated variable selection, providing a complementary safeguard against overfitting to spurious associations.

The potential influence of changes in concomitant preventive medication on treatment response will be explored through a sensitivity analysis rather than included as a fixed covariate in the primary predictive model, given the constraints imposed by the small sample size on the number of estimable parameters.

Patients who are lost to follow-up before the 12-month endpoint will be handled as follows: the primary analysis will be conducted on the per-protocol population (i.e., patients with complete 12-month outcome data). As a sensitivity analysis, patients lost to follow-up will be classified as non-responders to assess the impact of attrition on the results. Given the small expected sample size, every effort will be made to minimize losses to follow-up through active patient engagement and flexible scheduling of visits.

#### 2.5.2. Multidimensional Predictive Modeling

To address the primary objective of identifying predictors of response, we will develop a multivariate predictive model. We acknowledge the statistical challenge posed by the “curse of dimensionality,” where the number of measured biomarkers (predictors, p) exceeds the number of observations (participants, n). To mitigate the risk of overfitting inherent to standard logistic regression in this context, and to leverage the richness of the multidimensional biomarker panel (encompassing structural neuroimaging volumetrics, plasma peptide levels (CGRP, PACAP38, VIP), neurophysiological evoked potentials, and psychophysical algometry thresholds), we will employ penalized logistic regression with Elastic Net regularization.

This machine learning approach is specifically selected for its ability to:

1. Handle Multicollinearity: It effectively manages the high correlation expected between biological variables (e.g., between different pain thresholds or brain volumes) by combining the properties of Ridge (L2) and Lasso (L1) regression.
2. Perform Feature Selection: The algorithm will automatically shrink coefficients of non-informative variables to zero, effectively isolating the specific “biosignature” of responders from the noise.

The primary predictive model will be penalized logistic regression with Elastic Net regularization, implemented using the glmnet package in R. The mixing parameter (α) and the regularization strength (λ) will be optimized within a nested cross-validation framework. Specifically, the outer loop will consist of Leave-One-Out Cross-Validation (LOOCV) for unbiased performance estimation (see Section 2.5.3), while the inner loop will employ (N − 2)-fold cross-validation on the training subset of each outer iteration to select the optimal hyperparameters. As a complementary approach, a Random Forest classifier will be trained using the ranger package to capture potential non-linear relationships and higher-order interactions among biomarkers that may not be detected by a linear model. The Random Forest model will also provide variable importance rankings based on permutation accuracy, offering an additional perspective on the relative contribution of each biomarker domain. Given the limited sample size, variable importance rankings derived from the Random Forest should be interpreted as exploratory and complementary to the Elastic Net results, as permutation-based importance estimates may exhibit instability with fewer than 20 observations. Model performance will be compared using the cross-validated Area Under the Receiver Operating Characteristic Curve (AUC-ROC), sensitivity, specificity, and balanced accuracy. The final predictive panel will be derived from the model demonstrating the best cross-validated performance, with preference given to the more parsimonious model in cases of comparable discriminative ability.

#### 2.5.3. Validation Strategy

Given the limited sample size, internal validation will be performed using Leave-One-Out Cross-Validation (LOOCV). This iterative technique maximizes the training data by using N − 1 samples to train the model and the single remaining sample for validation, repeating the process N times. This approach provides a nearly unbiased estimate of the model’s prediction error and Area Under the Curve (AUC). To assess the stability of the selected predictors, we will report the selection frequency of each variable across the N LOOCV iterations (i.e., the proportion of folds in which each variable received a non-zero coefficient in the Elastic Net model). Variables selected in more than 80% of iterations will be considered stable predictors, whereas those with lower selection frequencies will be flagged as requiring confirmation in larger cohorts.

To facilitate interpretation and transparent reporting of the results, the following graphical outputs are pre-specified as part of the analytical plan: (a) Receiver Operating Characteristic (ROC) curves displaying sensitivity versus 1-specificity for each individual biomarker showing significant or near-significant bivariate associations with treatment response, as well as for the combined multivariable models (Elastic Net and Random Forest), enabling visual comparison of univariate versus multivariate discriminative performance; (b) a dot plot or bar chart of variable importance scores derived from the Elastic Net (absolute standardized coefficients) and Random Forest (permutation importance) models, to illustrate the relative contribution of each biomarker domain; (c) a calibration plot comparing predicted probabilities of response against observed response rates, acknowledging that calibration assessment will be interpreted with caution given the limited sample size and reported primarily for methodological completeness; and (d) a forest plot summarizing the odds ratios and 95% confidence intervals for variables retained in the final penalized model. Additionally, a CONSORT-style flowchart will be presented to document participant progression through each study period.

This comprehensive analytical strategy ensures that despite the limited cohort size, the depth of phenotyping allows for the hypothesis-generating identification of multivariate patterns that would be undetectable with univariate methods, thereby advancing toward a precision medicine model for rCCH.

#### 2.5.4. Use of AI-Assisted Tools

NotebookLM (Google, USA, powered by Gemini 3) was used exclusively as an assistive tool for the design of Figure 1 and the graphical abstract.

## 3. Results and Discussion

The findings of this study would represent an advance in the understanding of factors associated with favorable or unfavorable responses to ONS treatment. This would enable the identification of a biomarker or combination thereof, capable of reliably predicting patients who would benefit from this treatment modality.

This study is pioneering in its multidimensional approach to identifying predictors of response to ONS in patients with rCCH. To date, no study has systematically and prospectively identified predictive biomarkers in this population. A recent study examined the opposite premise [13], identifying clinical variables such as early onset, smoking, and seasonal or circadian variation as potential factors associated with ONS treatment failure. However, a subsequent subanalysis of the ICON study did not find these variables to be predictive factors for non-response [33]. Literature has thus far found no consistency among purely clinical factors that would allow for the precise identification of patients who would benefit from this therapy, which, although minimally invasive, is not without risks.

A critical confounding factor in the existing literature is the heterogeneity of surgical techniques and neuromodulation systems used across different cohorts, which often complicates the comparison of outcomes [34]. In contrast, a major strength of our study is the standardization of the surgical procedure; our center follows a defined and identical implantation protocol that has remained consistent for 15 years, aligned with established practices in other leading specialized units [35]. This methodological consistency reduces the risk of technical variance and ensures that differences in clinical outcomes are more likely attributable to the biological and psychological phenotypes of the patients themselves.

The primary strength of this study is its prospective design and comprehensive characterization of patients using a biomarker panel encompassing multiple domains: structural neuroimaging, neurophysiology, somatosensory pain processing, neuropsychological profile, and serum markers. The identification of potential predictive biomarkers of response could also provide insights into the pathophysiological mechanisms, thereby opening new avenues for research.

The research team has extensive experience in the management of patients with rCCH and has previously published one of the largest case series with the longest follow-up period of patients treated with ONS [32] and a systematic review and meta-analysis on the treatment of this condition [7]. Additionally, the CLUSTER-MAD registry provides data on the current treatment landscape in our setting [13].

Furthermore, the inclusion of TENS as a potential non-invasive predictor represents a clinically relevant contribution, as it could serve as an accessible screening tool prior to undergoing the surgical procedure [26,31].

The main limitation of this study is the small sample size, which is inherent to the low prevalence of rCCH. With an expected enrollment of approximately 15 patients, the ratio of predictors to observations poses a well-recognized statistical challenge, as overfitting becomes a substantial risk when the number of candidate biomarkers approaches or exceeds the sample size. Standard multivariable regression models are particularly vulnerable in this setting, as they may capture noise rather than true predictive signals, yielding optimistic performance estimates that fail to generalize. To mitigate this risk, we have adopted two complementary strategies. First, the use of Elastic Net regularization imposes a penalty on model coefficients, effectively shrinking non-informative variables toward zero and reducing model complexity. Second, Leave-One-Out Cross-Validation (LOOCV) provides a nearly unbiased estimate of prediction error by iteratively training the model on N − 1 observations and testing on the single held-out case. Unlike k-fold cross-validation, LOOCV maximizes the training set size at each iteration, which is critical when data are scarce. However, it should be acknowledged that LOOCV estimates can exhibit high variance because the N training sets overlap substantially, meaning each fold differs by only a single observation. Consequently, the resulting AUC and classification metrics should be interpreted as preliminary estimates that require external validation. Even within these constraints, we consider this approach appropriate for an exploratory study aimed at generating hypotheses and identifying candidate biomarker signatures to be confirmed in future larger, multicenter cohorts. Furthermore, the variable importance rankings obtained from the Random Forest model should be interpreted with particular caution, as permutation-based estimates are known to exhibit substantial instability in small samples. For the Elastic Net model, we will report the selection frequency of each predictor across LOOCV iterations as an indicator of stability, with the understanding that variables showing inconsistent selection may represent noise rather than true predictive signals. Nevertheless, even with a small sample size, the depth of multidimensional phenotyping may reveal clinically meaningful effect sizes and important trends that would be undetectable with univariate methods, thereby laying the groundwork for future multicenter validation studies.

Additionally, the absence of a sham-stimulated or untreated control group precludes definitive conclusions regarding whether the identified predictors are specific to ONS response or reflect a more general propensity to clinical improvement. However, the primary aim of this study is not to assess ONS efficacy—which has been demonstrated in prior randomized controlled trials [9,10]—but to identify baseline characteristics that differentiate responders from non-responders within the ONS-treated cohort. Future studies incorporating sham-controlled designs or external validation cohorts may help clarify the mechanistic specificity of the predictors identified herein.

Furthermore, the single-center design of this study may limit the generalizability of the findings, as patient characteristics, surgical techniques, and clinical management protocols may vary across institutions. However, it should be noted that our center functions as a tertiary referral unit for occipital nerve stimulation, with an estimated 50–60% of patients referred from outside our primary catchment area, encompassing diverse healthcare settings and geographic regions. This referral pattern broadens the demographic and clinical heterogeneity of the study population beyond what would be expected from a purely local cohort, partially mitigating the limitations typically associated with single-center designs. Nevertheless, although the standardization of the surgical procedure at our center reduces technical variance and strengthens internal validity, formal multicenter collaboration would be necessary to confirm the external validity and reproducibility of the identified predictors across different clinical settings and populations, while also improving recruitment rates for this low-prevalence condition.

Notwithstanding these limitations, the findings of this study could contribute to the adoption of a precision medicine strategy for rCCH. This approach would not only enable appropriate candidate selection while avoiding adverse effects, but would also optimize the use of healthcare resources by preventing the implantation of costly devices (approximately €28,000) in patients with a low likelihood of response.

## 4. Conclusions

Through a multidimensional assessment encompassing neuroimaging, neurophysiology, algometry, neuropsychological profile, and serum markers, this study aims to identify predictive factors of response to ONS treatment, thereby improving patient selection, optimizing healthcare resources, and advancing the understanding of treatment response mechanisms. If a reliable set of predictors is identified and subsequently validated in larger multicenter cohorts, the long-term goal would be to integrate these biomarkers into a structured clinical decision-making algorithm for ONS candidacy. In practice, such an algorithm could stratify patients into high-, intermediate-, and low-probability responder categories based on their individual biomarker profile, guiding clinicians through a stepwise evaluation process: patients with a favorable multidimensional profile would proceed directly to ONS implantation, those with an unfavorable profile would be redirected to alternative therapeutic strategies, and patients in the intermediate category could undergo additional screening with TENS as a non-invasive confirmatory step before a final surgical decision. This approach would represent a shift from the current trial-and-error paradigm toward a precision medicine framework for rCCH, reducing unnecessary surgical morbidity and optimizing the allocation of healthcare resources.

## Figures and Tables

**Table 1:** Summary of assessments performed at each study period.

Study Period	Assessment	Variables/Instruments
**Pre-ONS**	Screening visit	Demographics, medical history, headache characteristics (attack frequency, VAS intensity), prior and current treatments, eDiary provision, PROMs (EQ-5D, CHQ, HIT-6, ASC-12)
*(Weeks 0–8)*	Structural 3T brain MRI	T1, T2/FLAIR, SWI, DWI, sagittal 3D T1 (volumetric evaluation of gray and white matter)
	Blood analysis	CGRP, PACAP38, VIP (ELISA)
	Neuropsychological evaluation	MMPI-3, MASK-5, HADS, PCS (120 min)
	Auditory evoked potentials	Absolute latencies of Waves I, III, V; interpeak intervals (I–III, III–V, I–V); interaural latency differences
	Algometry	PPT (trigeminal and extratrigeminal regions), temporal summation (wind-up ratio), CPM (cold pressor)
	TENS	Gate control mode (80 Hz, 100 μs), 3 × 30 min/day
**ONS**	Trial electrode implantation	Provisional ONS per standardized neurosurgery protocol
	Response assessment	≥30% reduction in weekly attack frequency
*(Weeks 8–14)*	Definitive IPG implantation	If response criteria met at week 14
	eDiary (continuous)	Attack frequency, intensity, duration, rescue medication, adverse effects
	Clinical follow-up	Attack frequency (per day/week), intensity (VAS), duration, rescue medication use, adverse effects, PROMs (EQ-5D, CHQ, HIT-6, ASC-12, HADS, PGI-C)
**Post-ONS**	Algometry (3, 6, 12 months)	PPT (trigeminal and extratrigeminal regions), temporal summation, CPM
*(3, 6, 12 months)*	Blood analysis (3 months)	CGRP, PACAP38, VIP (ELISA)
	eDiary (continuous)	Attack frequency, intensity, duration, rescue medication, adverse effects

Abbreviations: ASC-12, Allodynia Symptom Checklist-12; CHQ, Cluster Headache Quality of Life questionnaire; CGRP, calcitonin gene-related peptide; CPM, conditioned pain modulation; DWI, diffusion-weighted imaging; eDiary, electronic headache diary; ELISA, enzyme-linked immunosorbent assay; EQ-5D, EuroQoL five-dimension questionnaire; FLAIR, fluid attenuation inversion recovery; HADS, Hospital Anxiety and Depression Scale; HIT-6, Headache Impact Test-6; IPG, implantable pulse generator; MASK-5, Millon Adolescent Clinical Inventory-5; MMPI-3, Minnesota Multiphasic Personality Inventory-3; MRI, magnetic resonance imaging; ONS, occipital nerve stimulation; PACAP38, pituitary adenylate cyclase-activating peptide 38; PCS, Pain Catastrophizing Scale; PGI-C, Patient Global Impression of Change; PPT, pressure pain threshold; PROMs, patient-reported outcome measures; SWI, susceptibility-weighted imaging; TENS, transcutaneous electrical nerve stimulation; VAS, visual analog scale; VIP, vasoactive intestinal peptide.

## Data Availability

The anonymized dataset supporting the findings of this study will be made available by the corresponding author upon reasonable request, subject to compliance with the ethical approval granted by the Research Ethics Committee of Hospital Universitario La Paz (PI-6215) and applicable data protection regulations (EU General Data Protection Regulation 2016/679). Data will not be deposited in a public repository due to the sensitive nature of clinical and neuroimaging information from a small patient cohort, which may pose re-identification risks even after anonymization. In accordance with the FAIR (Findable, Accessible, Interoperable, Reusable) data principles, the study protocol, variable definitions, and analytical code (R scripts) will be shared alongside the dataset to facilitate independent validation and reproducibility.

## Abbreviations

The following abbreviations are used in this manuscript:

AC-PC	anterior commissure–posterior commissure
ASC-12	Allodynia Symptom Checklist-12
AUC	area under the curve
BAER	brainstem auditory evoked responses
CCH	chronic cluster headache
CEIm	Comité Ético de Investigación con Medicamentos (Research Ethics Committee for Medicinal Products)
CGRP	calcitonin gene-related peptide
CH	cluster headache
CHQ	Cluster Headache Quality of Life questionnaire
CPM	conditioned pain modulation
DWI	diffusion-weighted imaging
eDiary	electronic headache diary
EHF	European Headache Federation
ELISA	enzyme-linked immunosorbent assay
EQ-5D	EuroQoL five-dimension questionnaire
FLAIR	fluid attenuation inversion recovery
FSL	FMRIB Software Library
HADS	Hospital Anxiety and Depression Scale
HIT-6	Headache Impact Test-6
IPG	implantable pulse generator
IQR	interquartile range
LOOCV	leave-one-out cross-validation
MASK-5	Millon Adolescent Clinical Inventory-5
MMPI-3	Minnesota Multiphasic Personality Inventory-3
MRI	magnetic resonance imaging
ONS	occipital nerve stimulation
PACAP38	pituitary adenylate cyclase-activating peptide 38
PCS	Pain Catastrophizing Scale
PGI-C	Patient Global Impression of Change
PPT	pressure pain threshold
rCCH	refractory chronic cluster headache
REDCap	Research Electronic Data Capture
SD	standard deviation
SWI	susceptibility-weighted imaging
TENS	transcutaneous electrical nerve stimulation
TSE	turbo spin echo
VAS	visual analog scale
VIP	vasoactive intestinal peptide
